# Severity of Chagasic Cardiomyopathy Is Associated With Response to a Novel Rapid Diagnostic Test for *Trypanosoma cruzi* TcII/V/VI

**DOI:** 10.1093/cid/ciy121

**Published:** 2018-02-09

**Authors:** Tapan Bhattacharyya, Louisa A Messenger, Caryn Bern, Pascal Mertens, Quentin Gilleman, Nicolas Zeippen, Bruno C Bremer Hinckel, Niamh Murphy, Robert H Gilman, Michael A Miles, Chagas Working Group in Bolivia and Peru, Jorge Flores, Roni Colanzi, Ricardo Bozo, Gerson Galdos, Manuela Verastegui, Gerardo Sanchez, Emi Okamoto, Jackie Sherbuck, Toria Rendell, Vishal Shah, Malasa Jois, Diana Marti

**Affiliations:** 1 Faculty of Infectious & Tropical Diseases, London School of Hygiene & Tropical Medicine, United Kingdom; 2 Department of Epidemiology and Biostatistics, School of Medicine, University of California, San Francisco; 3 Coris BioConcept, Gembloux, Belgium; 4 Department of Biomedical Sciences, University of Antwerp, Antwerp, Belgium; 5 Department of International Health, Johns Hopkins Bloomberg School of Public Health, Baltimore, Maryland

**Keywords:** Chagas disease, cardiomyopathy, prognosis, rapid diagnostic test, *Trypanosoma*, *cruzi*

## Abstract

**Background:**

*Trypanosoma cruzi* causes Chagas disease in the Americas. The outcome of infection ranges from lifelong asymptomatic status to severe disease. Relationship between *T. cruzi* lineage (TcI-TcVI) infection history and prognosis is not understood. We previously described peptide-based lineage-specific enzyme-linked immunosorbent assay (ELISA) with trypomastigote small surface antigen (TSSA).

**Methods:**

A novel rapid diagnostic test (RDT; Chagas Sero K-SeT) that incorporates a peptide that corresponds to the TSSA II/V/VI common epitope was developed and validated by comparison with ELISA. Patients from Bolivia and Peru, including individuals with varying cardiac pathology, and matched mothers and neonates, were then tested using Chagas Sero K-SeT.

**Results:**

Chagas Sero K-SeT and ELISA results, with a Bolivian subset of cardiac patients, mothers, and neonates, were in accord. In adult chronic infections (n = 121), comparison of severity class A (no evidence of Chagas cardiomyopathy) with class B (electrocardiogram suggestive of Chagas cardiomyopathy) and class C/D (decreased left ventricular ejection fraction; moderate/severe Chagas cardiomyopathy) revealed a statistically significant increase in Chagas Sero K-SeT reactivity with increasing severity (χ2 for trend, 7.39; *P* = .007). In Peru, Chagas Sero K-SeT detected the sporadic TcII/V/VI infections.

**Conclusions:**

We developed a low cost RDT that can replace ELISA for identification of TSSA II/V/VI immunoglobulin G. Most importantly, we show that response to this RDT is associated with severity of Chagas cardiomyopathy and thus may have prognostic value. Repeated challenge with T. cruzi infection may both exacerbate disease progression and boost the immune response to the TSSApep-II/V/VI epitope.

## Teaser

We describe a novel, point-of-care, rapid diagnostic test for *Trypanosoma cruzi* TcII/V/VI infection. Positive test response is associated with severity of Chagas cardiomyopathy. Repeated *T. cruzi* infection may exacerbate disease and boost immune response to this TSSApep-II/V/VI epitope.

*Trypanosoma cruzi* is the agent of Chagas disease, considered the most important human parasitic disease in Latin America. *Trypanosoma cruzi* is transmitted via contamination with infected feces of blood-feeding triatomine bugs through mucous membranes, abraded skin, or oral ingestion. The parasite can also be transmitted congenitally and by contaminated blood components or organs. The initial acute phase may be mild and unrecognized, but the disease can be fatal, particularly in immunocompromised hosts. The subsequent chronic phase may be asymptomatic for life, but around 30% of those infected progress to Chagas cardiomyopathy, and a smaller percentage may develop gastrointestinal megasyndromes. A recent World Health Organization report [1], based on data for 2010, estimates that approximately 5.7 million people in Latin America are infected with *T. cruzi*, of which Bolivia and Peru account for approximately 600000 and 120000 cases, respectively. Bolivia has the highest estimated number of new vector-borne infections and the highest prevalence.

*Trypanosoma cruzi* encompasses 6 intraspecies lineages (discrete typing units) TcI–TcVI, with a seventh proposed (TcBat) related to TcI [2]. TcI is distributed throughout the Americas and frequent among human cases in Mexico, Central America, and northern South America, whereas TcII, TcV, and TcVI principally circulate in the Southern Cone countries of South America. TcIII is widespread in wildlife in South America, including armadillos (*Dasypus*), but rare in humans. TcIV is the secondary agent of Chagas disease in Venezuela and, with TcI, commonly found in mammals in the United States [2–5].

Association between an individual’s history of *T. cruzi* lineage infection and clinical outcome remains an area of great research interest. For example, certain lineages might more frequently be associated with severe chronic disease, such as gastrointestinal manifestations [3, 6]. However, the sequestration and replication of *T. cruzi* in host tissues, low circulating parasitemias, and selection of isolates in cultures hamper the identification of representative lineages by direct genotyping of clinical samples.

There are many commercial and in-house diagnostic serological tests for *T. cruzi* infection, with diverse sensitivities and ease of use [7]. To date, the mucin trypomastigote small surface antigen (TSSA), expressed on the bloodstream trypomastigote form, has been the only *T. cruzi* antigen shown to be applicable to lineage-specific serology [8–10]. The protein core of TSSA contains remarkable polymorphism, in which TcI, TcIII, and TcIV have their own lineage-specific sequences. The TcII sequence is shared with TcV/VI except that 1 haplotype of the heterozygous TcV/VI allele is specific to these hybrid lineages [11]. Previously, we used synthetic peptides representing the lineage-specific TSSA epitopes as antigens for serology with sera of chronic Chagas disease patients from South American countries [9]. We observed that the peptide representing the TSSA epitope common to TcII/V/VI (TSSApep-II/V/VI) had a high level of recognition by immunoglobulin (Ig) G from patients in Southern Cone countries, including Bolivia. Furthermore, a higher proportion of TSSApep-II/V/VI seropositive patients from Brazil had electrocardiogram (ECG) abnormalities compared with patients who were TSSApep-II/V/VI seronegative [9].

Our objectives were to adapt TSSApep-II/V/VI–specific serology to a novel, low-cost, point-of-care, lateral flow immunochromatographic rapid diagnostic test (RDT); to compare the RDT to the lineage-specific peptide enzyme-linked immunosorbent assay (ELISA); and to apply the RDT to serology of clinically assessed *T. cruzi*-infected Bolivian and Peruvian patients previously untested by lineage-specific serology.

## METHODS

### Origin of Samples

The samples originated from previously reported studies [12–17]. *Trypanosoma cruzi* infection was defined by positive results by ≥2 conventional serological tests.

Set 1, Bolivia [12, 13]: comprised maternal and cord blood from Santa Cruz, a city without vector-borne *T. cruzi* transmission, and from the smaller city of Camiri not far from villages with triatomine infestation.

Set 2, Bolivia: samples were from a study of potential cardiac biomarkers in adults in Santa Cruz. Data included New York Heart Association (NYHA) class and cardiac severity stages A, B, C, and D, as defined by Okamoto et al [14]. All patients in stages C and D (defined by moderately and severely decreased left ventricular ejection fraction, analyzed together as C/D) had echocardiogram data. Those in NYHA class 0 (no restrictions on exercise tolerance) with normal ECG readings but no echo data were included in stage A (no evidence of cardiomyopathy); such individuals are considered unlikely to have significant abnormalities on ECG [18]. If the ECG reading showed abnormalities suggestive of Chagas cardiomyopathy, we included the patient in stage B. Some patients thus classified as stage B might have been assigned to stage C/D if the echo had been performed, but this misclassification would bias the cardiomyopathy analysis toward the null hypothesis. Participants without echo data and with ECG abnormalities not characteristic of Chagas cardiomyopathy were excluded from these analyses.

Set 3, Peru: specimens were from 2 studies in and near Arequipa in southern Peru [15, 16] and a community study in Cajamarca Department in northern Peru [17].

### Ethics

The following institutional review boards granted ethical approval:

Set 1. Johns Hopkins Bloomberg School of Public Health (JHBSPH, United States), Hospital Universitario Japonés (Bolivia), Universidad Católica Boliviana (UCB, Bolivia), Universidad Peruana Cayetano Heredia (UPCH, Peru), Asociación Benéfica Proyectos en Informatica, Salud, Medicina y Agricultura (A. B. PRISMA, Peru), and the Centers for Disease Control and Prevention (United States). Each woman provided written informed consent for herself and her infant.

Set 2. UCB, A. B. PRISMA, and JHBSPH. All participants provided written informed consent.

Set 3. Study [15]: JHBSPH, UPCH, University of Pennsylvania (Penn) Study [16]: JHBSPH, A.B. PRISMA Study [17]: A.B. PRISMA, Penn. All participants provided written informed consent.

Additional approval: serum collection and secondary data analysis was approved under the ChagasEpiNet protocol by the London School of Hygiene and Tropical Medicine.

### Lineage-Specific Peptide ELISA

Peptides TSSApep-II/V/VI, -III, -IV, and -V/VI representing residues 37–52 in the TSSA protein of those lineages and *T. cruzi* TcII lysate (IINF/PY/00/Chaco23cl4) were used in ELISA as described previously [9].

### Chagas Sero K-SeT RDT

**Figure 1.**
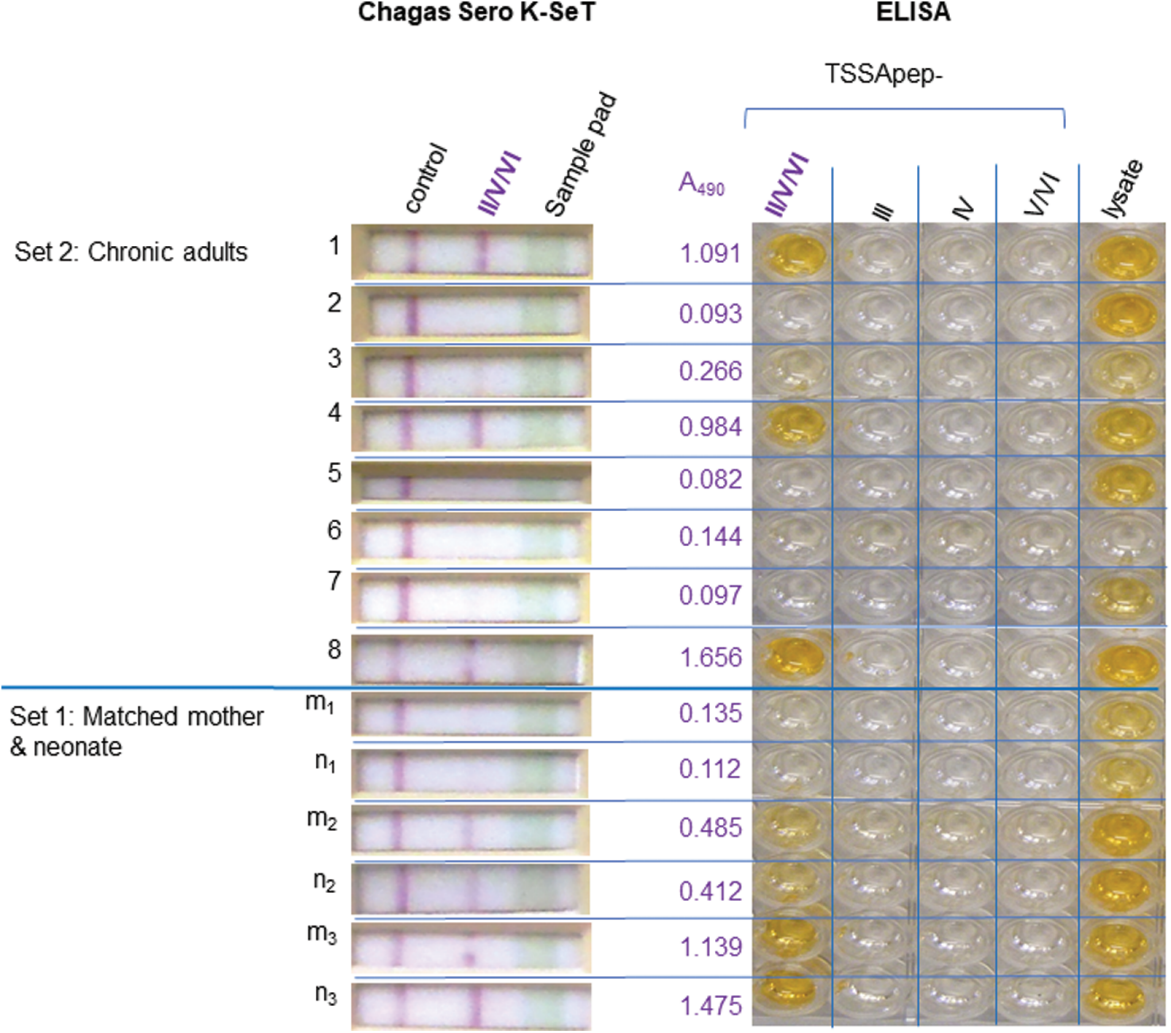
Concordance of Chagas Sero K-SeT and TSSApep enzyme-linked immunosorbent assay (ELISA). Representative samples from set 1 (matched mother–neonate) and set 2 (adult chronic infection) assayed using TSSApep ELISA and Chagas Sero K-SeT gave corresponding results for TSSApep-II/V/VI reaction, with 1 negative/borderline ELISA positive by rapid diagnostic text (pair m_1_ n_1_). Set 2, sample 6, was previously shown to be *Trypanosoma cruzi* seronegative. The A_490_ reading is for TSSApep-II/V/VI reactivity by ELISA and is the mean of duplicate assay plates.

This novel RDT was developed in the laboratories of Coris BioConcept. Plastic cassettes were constructed with a nitrocellulose membrane, sample, conjugate, and absorbent pads backed with a plastic strip. The cassette had 2 windows: a buffer application well and a test/reading window where the sample application zone is also located (Figure 1). The nitrocellulose membrane was sensitised with TSSApep-II/V/VI (GTENKPATGEAPSQPG) coupled to avidin. Depending on sample type, 2–3 µL of sample was applied to the sample application zone, then buffer was added in the buffer well. Immunoglobulin (IgG) antibodies from the sample that migrated over the nitrocellulose membrane reacted with the avidin-immobilized peptide. The protein G-gold conjugate rehydrated by the buffer recognized the TSSApep-II/V/VI–bound IgG, resulting in a red–purple colored band. A control line ensured that sample and conjugate migration had occurred. Tests were read after 15 minutes and scored as either positive (colored test and control lines) or negative (colored control line only) and photographed.

### Statistical Analysis

Categorical variables were compared using χ^2^ or Fisher exact test, as appropriate. Continuous variables were compared using the Wilcoxon ranked sum test.

## RESULTS

### Comparability of Lineage-Specific ELISA and Chagas Sero K-SeT RDT

A subset of samples from set 1 (5 matched pairs of maternal and cord blood) and set 2 (n = 15) were assayed using TSSApep lineage-specific ELISA and Chagas Sero K-SeT RDT. Of the samples in this subset, all from set 1 and 11 from set 2 were seropositive by conventional serology (CS+). Results from the 2 assays were concordant (Figure 1). Both assays detected maternal IgG transferred transplacentally. Thus, based on this subset of samples, the 2 lineage-specific assays, Chagas Sero K-SeT RDT and ELISA, performed at least equally well. Therefore, only Chagas Sero K-SeT RDT was used for the remaining samples.

### Set 1: Paired Maternal and Cord Blood

Specimens from 131 CS+ and 12 CS− women were tested. The mean maternal age was 27.6 years (standard deviation [SD] 7.2). RDT results were positive in 68.7% (57/83) and 66.7% (32/48) of CS+ mothers from Camiri and Santa Cruz, respectively (*P* = .81 for site comparison); however, there is active population migration at both sites. There was 100% concordance between maternal and cord blood by Chagas Sero K-SeT RDT (36 RDT positive and 21 RDT negative in both specimens). RDT results did not differ by infection status of the infant (4/6 infected vs 84/124 uninfected infants of infected mothers; *P* = 1.0 by Fisher exact) or by maternal age (*P* = .34). There was no significant difference in the frequency of positive RDT for the small number of women with ECG changes suggestive of Chagas cardiomyopathy compared with those with normal ECG (5/11 vs 65/95; *P* = .17 by Fisher exact).

### Set 2: Adult Chronic Infection

**Figure 2.**
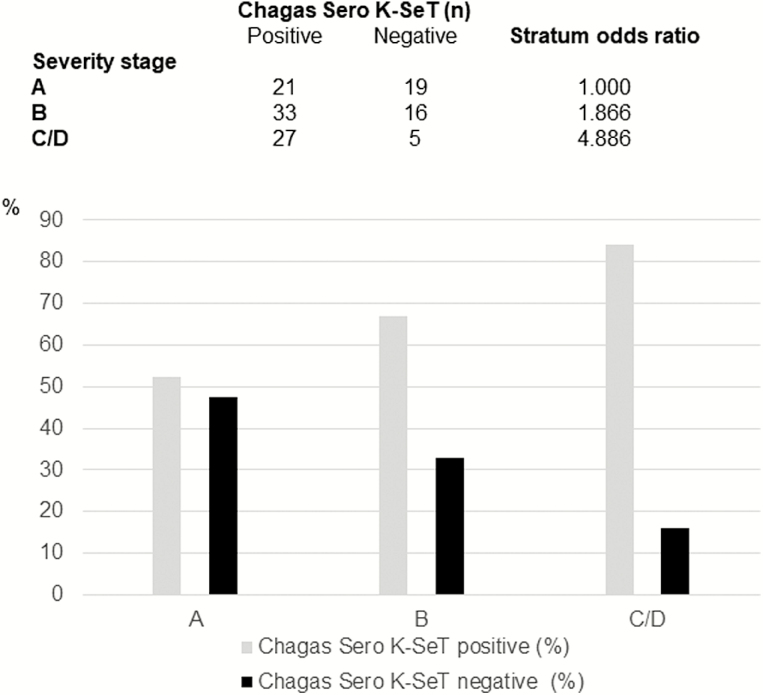
Association between Chagas cardiomyopathy severity and response to Chagas Sero K-SeT. Upper panel: cardiac severity stages (A–D) and Chagas Sero K-SeT result, where A = normal echocardiogram (ECG) plus normal left ventricular ejection fraction (LVEF) or New York Heart Association = 0 (in absence of echo), B = ECG abnormalities suggestive of Chagas cardiomyopathy, and C/D = decreased LVEF. Stratum odds ratio χ^2^ for trend 7.39; *P* = .007. Lower panel: graphical representation of these data.

A total of 121 CS+ and 27 CS− specimens were tested. Among CS+ patients, 73 (52.9%) were male and the mean age was 57.1 years (SD 12.3). CS− participants were significantly younger (mean age, 50.5 years; *P* = .008) and slightly less likely to be male (40.7% [11/27]; *P* = .29). There was no difference in the prevalence of positive RDT by participant age (*P* = .91) or sex (*P* = 1.0). However, 52.5% (21/40) of those without evidence of cardiomyopathy had positive RDT results compared with 74.1% (60/81) of those with cardiomyopathy (stage A vs stages B/C/D combined; *P* = .02). There was a significant trend for increasing frequency of RDT reactivity with increasing cardiac severity stage (*P* = .007 based on χ^2^ for trend; Figure 2).

### Set 3: Peruvian Specimens

A total of 84 specimens (65 CS+ and 19 CS−) were tested, 61 from southern Peru and 23 from northern Peru. The mean age of participants was 41.7 years (SD 19.8) and 59.5% were female. There was no significant difference in the prevalence of positive RDT in CS+ specimens from southern vs northern Peru (8.2% [4/49] vs 18.8% [3/16]; *P* = .35). However, the prevalence of positive RDT was markedly lower in Peru compared with Bolivia (10.8% [7/65] vs 66.9% [180/269]; *P* < .0001).

Of 58 CS−negative specimens, 2 (1 from Peru and 1 from Bolivia set 2) were detected by Chagas Sero K-SeT RDT as TcII/V/VI seropositive, corresponding to a specificity of 96.5% (95% confidence interval, 87.9–99.6) compared with conventional serology.

## DISCUSSION

The genetic lineages of *T. cruzi* are remarkably diverse. Understanding the relationship between an individual’s history of *T. cruzi* lineage infection and clinical outcome has been a research interest since the discovery of disparate geographical distributions of TcI and TcII between patients in Venezuela and Brazil, where prevalence of gastrointestinal syndromes also appeared strikingly divergent [3, 6]. Nevertheless, it is clear that Chagas cardiomyopathy occurs in TcI endemic regions and that TcI may cause severe disease in the immunocompromised [19]. Multiple potential confounders may complicate the association between *T. cruzi* diversity and clinical prognosis of Chagas disease, including human genetic diversity, immune competence, and exposure to coinfections [3]. Development of lineage-specific serology provides an alternative approach to studying the geography, epidemiology, and clinical relevance of the *T. cruzi* lineages, partially circumventing limitations of selective isolation and genotyping of *T. cruzi* strains.

The antigenicity of the TcII/V/VI isoform of TSSA has been established by serology of patients and animals in conjunction with bioinformatic and epitope mapping [8, 9, 20, 21]. Consistent with the absence of the TcII/V/VI epitope from all other *T. cruzi* lineage genomes, we demonstrated the serological specificity of TSSApep-II/V/VI and the absence of response in control infections [9]. However, because the sensitivity of TSSApep-II/V/VI serology is not 100%, seronegativity does not prove the absence of TcII/V/VI infection or that the agent of Chagas disease in such seronegative patients is TcI. Nevertheless, we have observed that the Chagas Sero K-SeT RDT consistently detected TSSApep-II/V/VI ELISA positives and, as noted here, occasional additional sera with negative/borderline ELISAs (Figure 1 [9], and unpublished data).

Previous lineage-specific serology with TSSA has used Western blot, ELISA, or other complex protocols that require skilled technical staff, laboratory facilities, and usually a cold storage chain [8–10]. We developed the Chagas Sero K-SeT RDT to overcome these practical and expensive issues, yet still allow for reliable and robust detection of IgG specifically recognizing TSSApep-II/V/VI. We show here that the Chagas Sero K-SeT RDT can replace ELISA for TSSApep-II/V/VI serology, detect lineage-specific IgG that is transferred across the placenta from an infected mother (set 1), is associated with higher likelihood of cardiac findings (set 2), and functions in geographical regions where some low anti-*T cruzi* antibodies have been reported (set 3).

To our knowledge, this is the first report of lineage-specific serology with matched maternal and cord blood. Bisio et al [22] found seropositivity to recombinant TSSA-II/V/VI among pregnant women from Argentina, Bolivia, and Paraguay but did not assess transplacental transfer of specific IgG. Genotyping of *T. cruzi* from neonates born to Bolivian mothers identified predominantly TcV; however, no association has been shown between *T. cruzi* lineage and congenital transmission risk [23]. Balouz et al [10] used recombinant TSSA-II/V/VI in serological assessment of treatment outcome in pediatric Chagas disease, indicating that this antigen could detect seronegativization sooner than conventional serological tests. While the Chagas Sero K-Set RDT does not solve the problem of diagnosing congenital infection at birth, its capacity to detect equal levels of anti-TSSA II/V/VI IgG in the mother and in cord blood illustrates its reliability, reproducibility, and wider potential as a consistent diagnostic assay.

Our analysis of results from older adults in Santa Cruz (set 2) demonstrates a significant association between the prevalence of positive results by this RDT and increasing severity of cardiomyopathy. We found evidence of a similar relationship in a previous study in southern Brazil, in which ECG abnormalities were more frequent among TSSApep-II/V/VI seropositive patients compared with TSSApep-II/V/VI seronegative patients [9]. Cytokine profiles of Chagas patients may be alternative signs of cardiomyopathy [14] but are not currently available as consistent indicators of prognosis or amenable to point-of-care rapid tests.

The mechanism underlying the association we observed between positive RDT and cardiac disease severity may be related to repeated parasite exposure over time. Investigators have previously postulated that repeated *T. cruzi* superinfection in those living in infested houses may increase inflammatory responses and the consequent risk of immunologically mediated cardiac damage [24]. Thus, repeated challenge with *T. cruzi* infection in triatomine-infested dwellings may both enhance the inflammatory response and boost the immune response to the TSSApep-II/V/VI epitope. Prolonged residence in an infested house was associated with increased risk of ECG abnormalities in our earlier analysis of data from Bolivian mothers [12].

We found a much lower prevalence of TcII/V/VI seropositivity among Peruvian patients than Bolivian patients. These results are in agreement with the widely reported low prevalence of these lineages outside the Southern Cone [2, 3, 25]. However, theoretically TcII/V/VI seropositivity might be partially influenced by reported low anti-*T. cruzi* immune responses among patients in southern Peru [16, 26]; even when rare, the TSSApep-II/V/VI RDT has the capacity to detect such infections.

Here, we have produced a highly informative, low-cost, point-of-care, lineage-specific RDT. Thus, there is justification to search for antigens applicable to each of the *T. cruzi* lineages, as well as for improved RDTs that are equally applicable across all diverse *T. cruzi* endemic regions. Furthermore, we have shown that the Chagas Sero K-Set RDT may provide an indicator of presence and risk of Chagas cardiomyopathy. We have proposed that this is explicable by concomitant exacerbation of disease and boosting of immune response to the TSSApep-II/V/VI epitope.

## Notes

***Acknowledgments.*** We thank collaborators in Bolivia, Peru, and the United States, including Jorge Flores, Roni Colanzi, Ricardo Bozo, Gerson Galdos, Manuela Verastegui, Gerardo Sanchez, Emi Okamoto, Jackie Sherbuck, Toria Rendell, Vishal Shah, Malasa Jois, and Diana Martin.

***Financial support.*** This work was supported by the National Institutes of Health (grant R01-AI087776, 5R01AI107028, D43TW006581). T. B. and M. A. M. have received support from the European Commission Framework Programme Project, Comparative Epidemiology of Genetic Lineages of *Trypanosoma cruzi*, ChagasEpiNet (contract 223034). B. C. B. was supported by European Commission Marie Sklodowska-Curie grant (agreement N642609).

***Potential conflicts of interest.*** All authors: No reported conflicts of interest. All authors have submitted the ICMJE Form for Disclosure of Potential Conflicts of Interest. Conflicts that the editors consider relevant to the content of the manuscript have been disclosed.
